# Sialic acids on T cells are crucial for their maintenance and survival

**DOI:** 10.3389/fimmu.2024.1359494

**Published:** 2024-06-14

**Authors:** Michael Schmidt, Alexandra T. Linder, Marina Korn, Nick Schellenberg, Sarah J. Meyer, Falk Nimmerjahn, Anja Werner, Markus Abeln, Rita Gerardy-Schahn, Anja K. Münster-Kühnel, Lars Nitschke

**Affiliations:** ^1^ Division of Genetics, Department of Biology, University of Erlangen, Erlangen, Germany; ^2^ Institute of Clinical Biochemistry, Hannover Medical School, Hannover, Germany

**Keywords:** sialic acids, T cell development, T cell activation, complement, apoptosis

## Abstract

Sialic acids are found as terminal sugars on glycan structures on cellular surfaces. T cells carry these sialoglycans abundantly, and they are thought to serve multiple functions in cell adhesion, cell migration, and protection from complement attack. We studied the role of sialoglycans on T cells in a mouse model with a T cell-specific deletion of cytidine monophosphate-sialic acid synthase (CMAS), the enzyme that is crucial for the synthesis of sialoglycans. These mice showed a T-cell deficiency in peripheral lymphoid organs. Many T cells with an undeleted *Cmas* allele were found in the periphery, suggesting that they escaped the Cre-mediated deletion. The remaining peripheral T cells of T cell-specific *Cmas* KO mice had a memory-like phenotype. Additional depletion of the complement factor C3 could not rescue the phenotype, showing that the T-cell defect was not caused by a host complement activity. *Cmas*-deficient T cells showed a high level of activated caspase 3, indicating an ongoing apoptosis. In bone marrow chimeric cellular transfer experiments, we observed a strong competitive disadvantage of *Cmas*-deficient T cells compared to wild-type T cells. These results show that sialoglycans on the surface of T cells are crucial for T-cell survival and maintenance. This function has not been recognized before and is similar to the function of sialoglycans on B cells.

## Introduction

T lymphocytes, like other mammalian cells, are covered with a dense layer of glycans, the so-called glycocalyx. The glycocalyx is built from several classes of monosaccharides in different glycosidic linkages, which creates a rich variation of glycan structures, attached to both proteins and lipids of the plasma membrane (1). In most cases, the glycan structures carry the terminal carbohydrate sialic acid (Sia) as a terminal sugar, forming the so-called sialoglycans. Due to the outermost position, Sia has important roles in cell–cell adhesion, cell migration, and cellular signaling (2). Sia is a ligand for sialic acid-binding immunoglobulin-like lectins (Siglecs), which are inhibitory receptors in immune cells and thereby regulate immune responses (3). Sia also forms ligands for selectins, receptors that regulate cell migration. Furthermore, sialoglycans can inhibit the complement system, as they can be bound by complement factor H (4). By this interaction, Sia contributes to discrimination between host and pathogen and protects host cells from attack by the complement system.

T lymphocytes typically carry Sia in α2,3- or α2,6-glycosidic linkage to either galactose or *N*-acetylgalactosamine and less frequently in α2,8 linkage to other Sia molecules. Specific sialyltransferases are required to transfer Sia onto glycans, creating sialoglycan structures. The enzymatic reactions of acceptor- and linkage-specific sialyltransferases require the activation of Sia to its cytidine-5′-monophospho-diester (CMP)-Sia form that is generated by the nuclear enzyme CMP-sialic acid synthase (CMAS) (5). *Cmas*-deficient mice that lack all sialoglycans on cellular surfaces die *in utero* at embryonic day E9.5. This embryonic lethality is caused by a maternal complement attack against fetal trophoblast cells that lack sialoglycans in *Cmas* ^−/−^ embryos and subsequently by defective development of extraembryonic tissues (6). In wild-type mice, protection from maternal complement attack is most likely achieved by complement factor H binding to α2,3-linked Sia on trophoblasts and inactivating maternal complement. Recently, we generated a B cell-specific *Cmas*-deficient mouse line that lacks all sialoglycans on B cells. Unexpectedly, these mice showed a severe B-cell deficiency in secondary lymphoid organs (7). B cells of B cell-specific *Cmas*-deficient mice showed impaired upregulation of the BAFF receptor, which is an important survival receptor. Furthermore, these B cells showed high levels of activated caspase 3 and cleaved caspase 8, indicating ongoing apoptosis. It was concluded from this study that sialoglycans on B cells are crucial for B-cell survival by protecting from several death-inducing pathways (7).

The global function of sialoglycans on T cells has not been studied so far. T cells develop in the thymus and are derived from precursor cells that migrate from the bone marrow to the thymus. Thymocytes develop in close contact with the thymic epithelial cells. Their first developmental stages take place in the thymic cortex, where the thymocytes are first double-negative (DN), referring to the absence of both CD4 and CD8 surface proteins. In the DN stage, they start rearranging their TCR genes, leading to a full TCR in the double-positive (DP) CD4^+^ CD8^+^ stage. In this stage, they are positively selected by binding to their own MHC proteins on epithelial cells, either MHC class I or MHC class II. Then, a negative selection takes place at the border of the cortex and medulla of the thymus, deleting those thymocytes that recognize self-peptides presented by MHC with a high affinity. In this way, the TCR repertoire is formed, and either single-positive (SP) CD4^+^ or SP CD8^+^ T cells leave the thymus through the medulla and migrate to peripheral lymphoid organs via the bloodstream. The changes in glycan composition during development and activation of T cells have been described in detail recently (8, 9). The deletion of specific sialyltransferases that are involved in the generation of α2,3-linked sialoglycans affected T-cell differentiation and selection differently. A deletion of the gene coding for the sialyltransferase ST3Gal-I led to severely reduced numbers of peripheral CD8^+^ T cells without affecting CD4^+^ T cells (10). This was mechanistically explained by ongoing apoptotic processes of CD8^+^ T cells in these mice. In contrast, the deletion of ST3Gal-IV or ST3Gal-VI enzymes, either alone or in combination, did not affect the total number of T cells (11). The same holds true for the main enzyme that creates α2,6-linked sialoglycans, ST6Gal-I. The deletion of this enzyme in mice affected B-cell functions, but consequences for T-cell differentiation or T-cell functions were not detected (12). As there may be redundant functions of the various enzymes that create α2,3-linked or α2,6-linked Sia on T-cell glycans, a general function of sialoglycans on T cells cannot be concluded from these studies.

Siglecs are Sia binding regulatory receptors that often act inhibitory on activating pathways in immune cells. These regulatory processes of Siglecs are often controlled by Sia-containing ligands *in cis* on the same cellular surface. While B cells and myeloid immune cell types all express specific Siglec proteins, this is generally not the case for T cells. None of the Siglec proteins is expressed in T cells, neither in humans nor in mice (3, 13). Therefore, Sia expression on T cells must have more general functions than regulation of signaling. We decided to delete all sialoglycans from the surface of T cells by crossing the *Cmas* ^fl/fl^ mouse line with the T cell-specific lck-cre mouse line. The lck-cre line expresses the Cre protein from the DN stage onward in the thymus (14). The consequences of the *Cmas* deletion in T cells for T-cell differentiation and T-cell survival are presented in this study.

## Materials and methods

### Mice

*Cmas* ^fl/fl^ mice (15) were crossed with lck^cre/+^ mice (14) to generate T cell-specific *Cmas* KO mice. Complement-deficient *C3* ^−/−^ mice (16) were crossed with *Cmas* ^fl/fl^ mice × lck^cre/+^ mice.

*CD45.1* congenic mice (17) were kindly provided by Thomas Winkler, University of Erlangen, Erlangen, Germany. All mouse lines were maintained on a C57BL/6 background. Experiments were performed in accordance with the German Animal Welfare Act and after approval by the Animal Welfare Committee.

### Cell preparation for flow cytometry

Cell preparation for flow cytometry single-cell suspensions of bone marrow, thymus, spleen, and lymph nodes was prepared in phosphate-buffered saline (PBS) (Life Technologies, Carlsbad, CA, USA). Erythrocyte depletion was conducted using an ACK lysis buffer.

### Extracellular staining

Cells were stained for 25 min at 4°C with antibodies (Abs), diluted in PBS containing 1% bovine serum albumin (BSA) (Roth, Karlsruhe, Germany), 2 mM ethylenediaminetetraacetic acid (EDTA) (Roth), and 0.01% sodium azide (Sigma-Aldrich, Darmstadt, Germany). The following Abs were used: anti-CD4 (clone: GK1.5; BD Pharmingen, San Diego, CA, USA), anti-CD8 (clone: 53–6.7; BioLegend, San Diego, CA, USA), anti-TCRβ (clone: H57–597; BioLegend), anti-TCRγδ (clone: eBioGL3; eBioscience, San Diego, CA, USA), anti-CD44 (clone: IM7; eBioscience), anti-CD62L (clone: MEL-14; eBioscience), anti-CD45.1 (clone: A20; BD Biosciences, San Jose, CA, USA), anti-CD45.2 (clone: 104; BioLegend), anti-B220 (clone RA3–6B2; BioLegend), anti-IgM (clone II/41; eBioscience), anti-IgD (clone 11–26c; BioLegend), anti-CD25 (clone PC61; BioLegend), and anti-CD3e (clone145–2C11; eBioscience). Fc-block (2.4G2; hybridoma generated in-house) was added to prevent unspecific binding via the Fc-part of the Abs to the cell’s Fc receptors. To stain dead cells, fixable viability dye eFL506 (eBioscience) was used. Biotinylated Abs were detected by an additional staining of the cells with streptavidin fluorochrome conjugations. Cells were washed and diluted in PBS containing 0.1% BSA, 2 mM EDTA, and 0.01% sodium azide for flow cytometry via CytoFLEX S (Beckman Coulter, Brea, CA, USA).

### Intracellular staining

For intracellular activated caspase 3 staining, cells were incubated for 10 min at room temperature to allow spontaneous apoptosis. Subsequently, extracellular staining was performed as described above. Cells were fixed and permeabilized with a Cytofix/Cytoperm kit (BD Biosciences) according to the manual, and intracellular staining was conducted using the Ab anti-activated caspase 3 (clone C92–605; BD Biosciences). For the identification of regulatory T cells, manual and reagents of the anti-Mouse FoxP3 Staining Set (eBioscience) were used. After an extracellular staining as described above, thymus and splenic cells were fixed and permeabilized according to protocol. The cells were then intracellularly blocked and stained with Fc-block and anti-Foxp3 (clone: FJK-16s; eBioscience). After a final wash step, the cells were resuspended in 1× Permeabilization Buffer and analyzed via CytoFLEX S (Beckman Coulter).

### Lectin staining

For glycosylation analysis, cells were first extracellularly stained as described above. Then, the cells were fixed with 2% paraformaldehyde and stained with biotinylated lectins from VectorLabs [Newark, CA, USA; *Sambucus nigra agglutinin* (SNA), *Maackia amurensis* agglutinin (MAA II), *Erythrina cristagalli* agglutinin (ECA), and peanut agglutinin (PNA)] for 20 min at 4°C. Subsequently, cells were washed and incubated with streptavidin conjugates for 20 min at 4°C.

### Genomic detection of CMAS deletion via PCR

Thymus and splenic T cells and non-T cells were sorted via AriaII (BD Biosciences). The sorted cells were then lysed with ddH_2_O 0.3 µg/µL Proteinase K overnight at 56°C. After the inactivation of the enzyme by incubation at 94°C for 10 min, a PCR for the deleted *CMAS* exon 4 allele was performed. The following primers were used: BWF59 fwd (5′-AGCGCCTGTGTACCCCTCTTA-3′), BWB58 rev (5′-GCGAGCAGCAAGTGAGCA-3′), and AMB40 rev (5′-TCAAGTTCAGAGGCTCAGTCACTTCACG-3′).

The amplified DNA fragments were loaded onto a 2% agarose gel.

### Calcium mobilization assays

Thymus cells of 1 × 10^7^ were resuspended in 700 µL of Roswell Park Memorial Institute (RPMI) media (Life Technologies) containing 5% fetal calf serum (FCS) (PAN-Biotech, Aidenbach, Germany) and loaded with 1.5 mM Indo-1 AM plus 15% pluronic acid F-127 (Molecular Probes, Eugene, OR, USA) by shaking for 25 min at 30°C. Afterward, 700 µL of RPMI media (Life Technologies) containing 10% FCS (PAN-Biotech) was added, and cells were incubated by shaking for another 10 min at 37°C. Cells were washed twice and extracellularly stained with anti-CD4, anti-CD8, and an unconjugated hamster IgG anti-TCRβ antibody as described above. Cells were washed, resuspended in Krebs-Ringer solution (DeltaSelect, Munich, Germany), and incubated at 37°C for 3 min, and calcium mobilization was measured via LSRII (Becton Dickinson, Franklin Lakes, NJ, USA). The baseline Ca^2+^ level was determined for 50 s. Stimulation of the TCR was conducted by crosslinking TCRs with 3.75 or 7.5 µg/mL anti-hamster IgG (Jackson ImmunoResearch, West Grove, PA, USA), and Ca^2+^ mobilization was measured for another 2 min. Loading efficiency was determined by stimulation with ionomycin (Sigma-Aldrich).

### TCRβ chain repertoire analysis

The TCRβ chain repertoire of thymus and splenic T cells was performed by extracellularly staining as described above. The used antibodies were anti-CD3, anti-CD4, anti-CD8, and antibodies from the Mouse Vβ TCR Screening Panel (Vβ 2, 3, 4, 5.1 and 5.2, 6, 7, 8.1 and 8.2, 8.3, 9, 10b, 11, 12, 13, 14, and 17a; BD Pharmingen).

### Generation of mixed bone marrow chimera

Recipient CD45.1 wt mice were sublethally irradiated with 9 Gy. After 24 h, they were intravenously reconstituted with a bone marrow mixture from CD45.1 wt donor mice (10%) and CD45.2 lck^cre/+^xCMAS^fl/fl^ donor mice (90%). Seven weeks later, the mice were sacrificed and extracellularly stained for B-cell markers (B220 and IgM) and T-cell markers (CD3, CD4, CD8, and TCRγδ) CD45.1 and CD45.2 as described above.

### Statistical analysis

Statistical analyses were performed using GraphPad Prism software. Unpaired Mann–Whitney *U*-test was used to evaluate significance, which was plotted only for the relevant data. Statistical data are presented as mean ± SD.

## Results

### T cell-specific *Cmas* KO mice have a T-cell deficiency in the periphery

**Figure 1:**
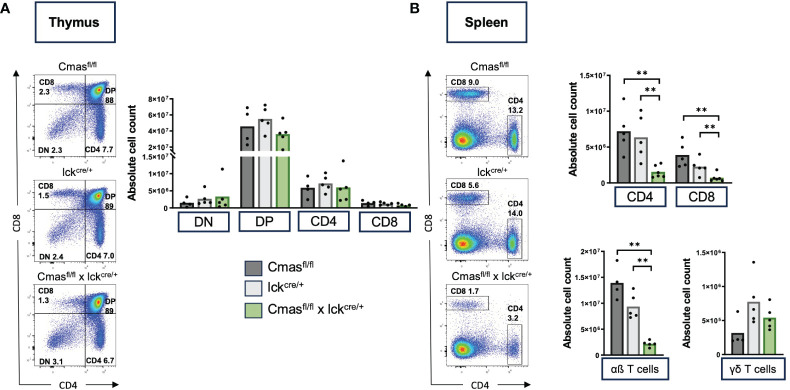
T cell-specific *Cmas* KO mice have strongly reduced T-cell numbers in the spleen. *Cmas* ^fl/fl^ × lck^cre/+^ mice and two control mouse lines (*Cmas* ^fl/fl^ and lck^cre/+^) were analyzed by flow cytometry for T-cell development. **(A)** CD4 versus CD8 staining shows unchanged numbers of double-negative (DN), double-positive (DP), or single-positive CD4^+^ or CD8^+^ T cells in the thymus of *Cmas* ^fl/fl^ × lck^cre/+^ mice. Left: representative staining is shown. Right: quantitative analysis of cell numbers. **(B)** CD4 versus CD8 staining shows a strong reduction of both CD4^+^ and CD8^+^ T cells in the spleen of *Cmas* ^fl/fl^ × lck^cre/+^ mice. Just the αβ T cells (CD3^+^ TCRβ^+^ TCRγδ^−^) were affected by the reduction, not the γδ T cells (CD3^+^ TCRβ^−^ TCRγδ^+^). Left: representative staining is shown. Right: quantitative analysis of cell numbers. N = 4–5 mice per group. Symbols represent individual animals. Data are representative of five individual experiments, each with one lck^cre/+^ and *Cmas* ^fl/fl^ × lck^cre/+^ mouse. In four of the experiments, a *Cmas* ^fl/fl^ control mouse was included. **p ≤ 0.01 (Mann–Whitney *U*-test).

We generated T cell-specific *Cmas* KO mice by crossing the Cmas^fl/fl^ mice (15) with lck-cre transgenic mice. The lck-cre line is a transgenic mouse line in which the cre is driven by the proximal lck promoter that is turned on in the DN stage of T-cell development in the thymus. It was reported that the cre expression in this mouse line affects T-cell development on its own (14). We therefore used for most of our experiments two control mice: *Cmas* ^fl/fl^ mice and lck-cre mice (with a wild-type *Cmas* gene). We indeed detected a mild reduction of peripheral αβ T-cell numbers in mice where the lck-cre allele was present on its own (**Figure 1B**). In the thymus of T cell-specific *Cmas* KO mice, we did not observe any changes in DN, DP, or SP T-cell populations, when compared to the two controls (**Figure 1A**). In the spleen, however, we found a reduction of CD4^+^ and CD8^+^ T cells when compared to both controls (**Figure 1B**). A similar, even stronger reduction of CD4^+^ and CD8^+^ T cells was observed in the lymph nodes of T cell-specific *Cmas* KO mice (**Supplementary Figure 1**). The T-cell deficiency specifically affected the classical αβ T cells, but not the γδ T cells, which showed rather higher numbers in the spleen (**Figure 1B**). This phenotype can be attributed to the presence of the lck-cre allele, as it was described before that in this transgenic line, γδ T cells are elevated in relative proportion, when compared to αβ T cells (18).

**Figure 2:**
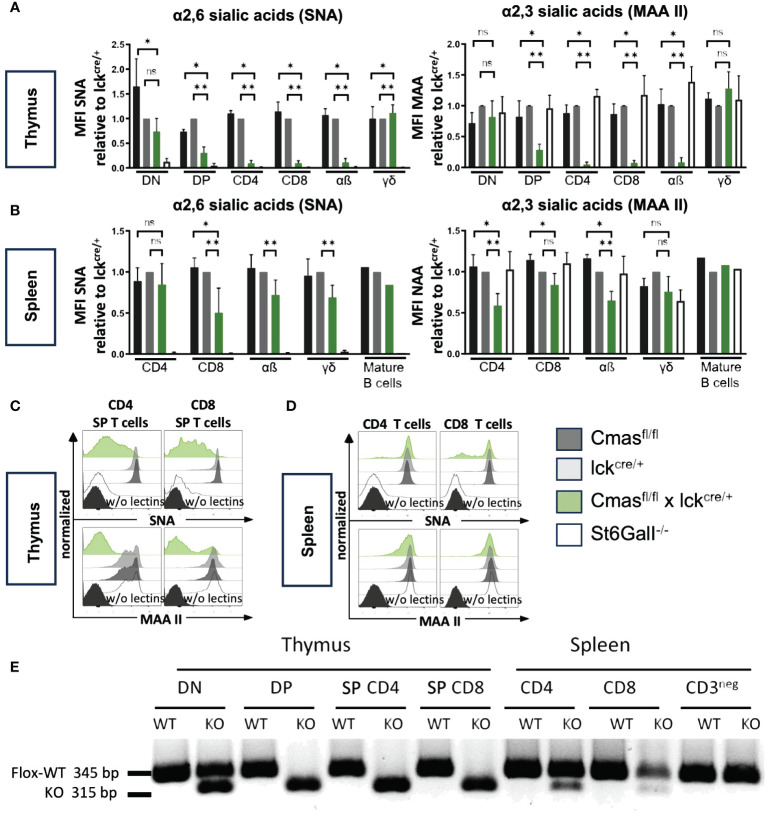
*Cmas* deficiency leads to a loss of sialic acid (Sia) on T cells in the thymus, but not in the spleen, due to incomplete *Cmas* deletion. **(A)** α2,6-Linked Sia and α2,3-linked Sia on the cellular surface of thymocytes were detected by lectin staining with the linkage-specific lectins *Sambucus nigra* agglutinin (SNA) and *Maackia amurensis* agglutinin (MAA II), respectively. To illustrate the reduction of surface sialylation during T-cell development in the *Cmas* ^fl/fl^ × lck^cre/+^ mouse compared to the lck^cre/+^ control mouse, the mean fluorescence intensity (MFI) was normalized by ratio to the MFI of the lck^cre/+^ control in each experiment (set as 1.0). ST6Gal-I^−/−^ mice were included as a control with no α2,6-linked Sia expression. **(B)** α2,6-Linked Sia and α2,3-linked Sia expression was detected with the same lectins for T cells in the spleen. The downregulation of Sia from the surface of CD4^+^ or CD8^+^ T cells is only weakly detected in the spleen of *Cmas* ^fl/fl^ × lck^cre/+^ mice. N = 4–5 mice per group. Data in panels **(A, B)** are representative of five individual experiments, each with one lck^cre/+^, *Cmas* ^fl/fl^ × lck^cre/+^, and ST6Gal-I^−/−^ mouse. In four of the experiments, a *Cmas* ^fl/fl^ control mouse was included. As a control for the T-cell specificity of the CRE-Recombinase, the sialylation of mature B cells was also analyzed in one of the experiments. *p ≤ 0.05, **p ≤ 0.01, ns = not significant (Mann–Whitney *U*-test). **(C, D)** Representative histograms show individual lectin staining examples for thymus T cells or splenic T cells, respectively. The same unstained controls (FMO w/o lectins) were used for SNA and MAA II staining in panels **(C)** and **(D)**. **(E)** Sorted double-negative (DN), double-positive (DP), or single-positive (SP) T cells from the thymus or CD4^+^ or CD8^+^ T cells from the spleen were tested by PCR for the amount of the floxed or the deleted (KO) *Cmas* allele. Data are representative of two individual experiments.

We performed lectin staining by flow cytometry to determine the degree of loss of Sia on different T-cell populations of T cell-specific *Cmas* KO mice. For this analysis, T-cell subpopulations of *Cmas* ^fl/fl^, *Cmas* ^wt^ × lck-cre, and *Cmas* ^fl/fl^ × lck-cre mice were stained either with SNA, specific for α2,6-linked Sia or with MAA II binding α2,3-linked Sia. We observed downregulation of both α2,6-linked Sia and α2,3-linked Sia during T-cell differentiation in the thymus of T cell-specific *Cmas* KO mice (**Figures 2A, C**). While DN cells still expressed near normal levels of sialoglycans, cell surface Sia was increasingly lost in the DP stage. SP CD4^+^ or CD8^+^ thymic T cells of T cell-specific *Cmas* KO mice had lost almost all α2,6-linked Sia, and thus resembled a control mouse with an ST6Gal-I deficiency, the sialyltransferase responsible for this linkage (12). *Cmas* KO SP T cells additionally lost α2,3-linked Sia (**Figures 2A, C**). The loss of sialoglycans in the thymus affected only SP αβ T cells, but not γδ T cells (**Figure 2A**). It is known that the proximal lck promoter is not effective in γδ T cells, therefore these cells are spared from cre deletion of target genes (18).

When we performed similar lectin staining on CD4^+^ and CD8^+^ T cells in the spleen of T cell-specific *Cmas* KO mice, we surprisingly found only a very minor reduction of both α2,6-linked Sia and α2,3-linked Sia. The mean reduction of Sia on splenic T cells was between 0 and 50% (**Figure 2B**). The primary lectin staining showed sometimes two populations, one with wild-type levels of Sia, and another one with downregulation Sia (**Figure 2D**). This indicated heterogeneous populations. We also used the lectins ECA and PNA recognizing terminal β1,4-linked galactose or β1,3-linked galactose, respectively. In wild-type T cells, most galactose residues are masked by a terminal sialic acid and therefore cannot be bound by these lectins. Accordingly, we observed increased ECA and PNA binding on SP T cells in the thymus of T cell-specific *Cmas* KO mice, but not on control T cells (**Supplementary Figure 2A**). In contrast, ECA and PNA binding was only weakly increased on splenic T cells of T cell-specific *Cmas* KO mice (**Supplementary Figure 2B**).

In order to find out whether the genetic deletion of *Cmas* by the lck-cre is complete, DN, DP, and SP CD4^+^ or CD8^+^ T cells from the thymus or CD4^+^ or CD8^+^ T cells and CD3^−^ non-T cells were sorted and tested by a genomic PCR that detects the Cre-mediated deletion of exon 4 of the *Cmas* gene. As expected, the deletion was detected at approximately 50% in the DN stage of T cell-specific *Cmas* KO mice and at approximately 100% in the DP and SP T-cell stages of the thymus. However, only a minor *Cmas* exon 4 deletion was detected in CD4^+^ and CD8^+^ T cells of the spleen (**Figure 2E**). In the spleen of T cell-specific *Cmas* KO mice, most T cells carried a *Cmas* wild-type allele. This indicated a leakiness of the lck-cre-mediated *Cmas* deletion in the thymus and a strong selection of WT T cells with normal sialoglycan levels into the spleen.

### T cells of T cell-specific *Cmas* KO mice have an activated phenotype, but a normal TCR repertoire

**Figure 3:**
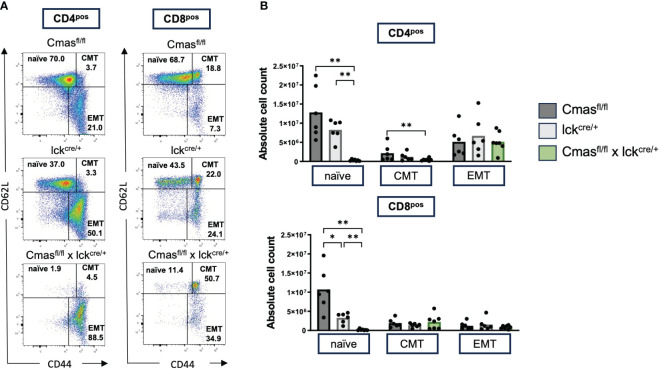
Splenic T cells of T cell-specific *Cmas* KO mice show a memory-like phenotype. Splenic CD4^+^ or CD8^+^ T cells were stained with CD62L and CD44 to determine the relative proportion of naïve, central memory (CMT), and effector memory (EMT) T-cell populations as indicated in panel **(A)** as representative dot plots. The cellular populations are quantified in panel **(B)** n = 6–7 mice per group. Symbols represent individual animals. Data are representative of seven individual experiments, each with one *Cmas* ^fl/fl^ × lck^cre/+^ mouse. In two of the experiments, only one control mouse was included. In one is the *Cmas* ^fl/fl^ control and in the other the lck^cre/+^ control. *p ≤ 0.05, **p ≤ 0.01 (Mann–Whitney *U*-test).

**Figure 4:**
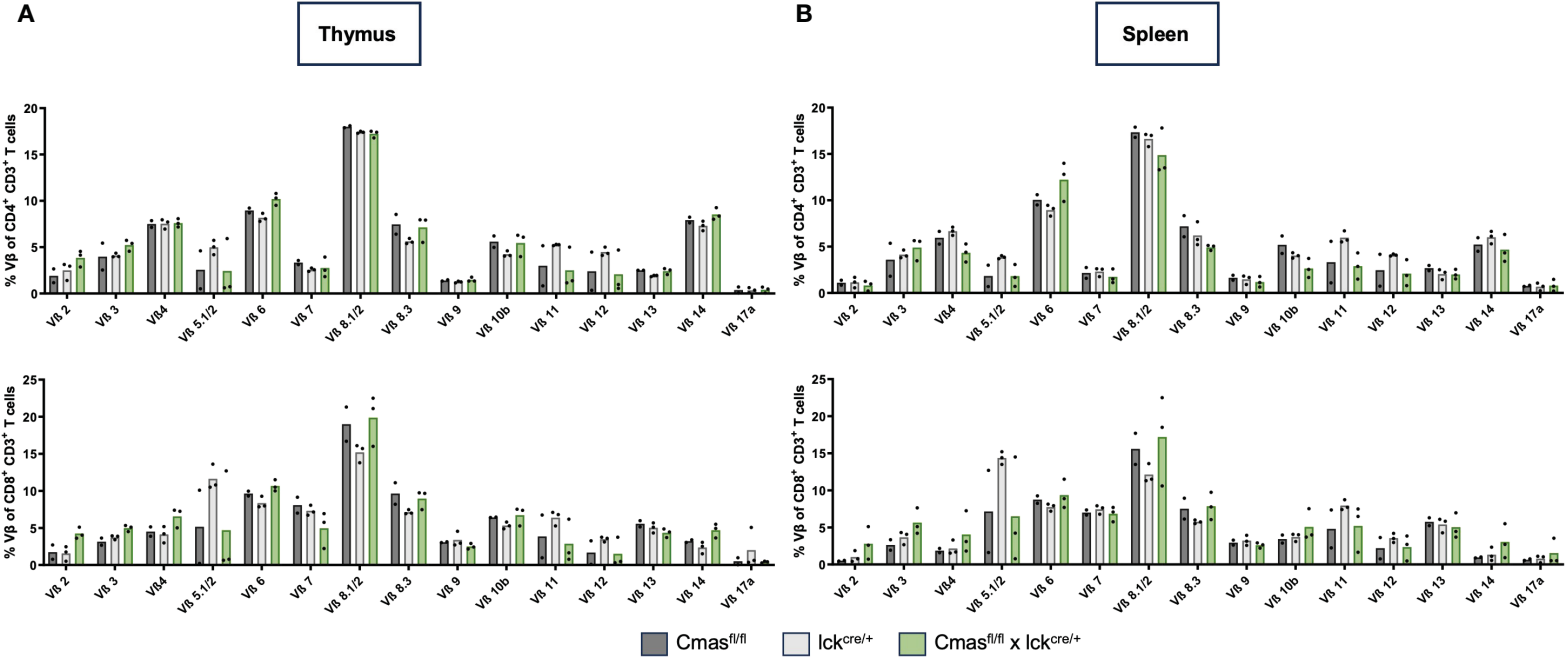
T cells of T cell-specific *Cmas* KO mice show a normal Vβ TCR repertoire. T cells (CD4^+^ CD3^+^ or CD8^+^ CD3^+^) of the thymus **(A)** or of the spleen **(B)** were stained with a panel of Vβ-specific antibodies. Bar diagrams show percentages of the respective Vβ expression. n = 2–3 mice per group. Data are representative of three individual experiments, each with one lck^cre/+^ and *Cmas* ^fl/fl^ × lck^cre/+^ mouse. In two of the experiments, a *Cmas* ^fl/fl^ control mouse was included.

From the lectin staining and genomic PCR analysis, we concluded that the peripheral T-cell repertoire of T cell-specific *Cmas* KO mice is a mixture of Sia-deficient and wild-type T cells that are preferentially selected. We determined the composition of naïve versus effector and memory T cells with CD62L/CD44 staining. We observed a strong reduction of naive T cells but normal numbers of central memory and effector memory-like T cells in T cell-specific *Cmas* KO mice (**Figure 3**). The higher proportion of memory and effector memory T cells in these mice may indicate a selection of certain T-cell subpopulations into the periphery, which could potentially affect the TCR repertoire of the remaining T cells in the periphery. In order to exclude this, the TCR repertoire of thymic and splenic T cells was determined by staining with a panel of Vβ-specific antibodies. We did not detect any differences in the TCR Vβ repertoire in the CD4^+^ or CD8^+^ T-cell population in the thymus or spleen of T cell-specific *Cmas* KO mice, compared to the two control mouse lines (**Figure 4**). We furthermore observed that in both the thymus and spleen, the total number of regulatory T cells was reduced in T cell-specific *Cmas* KO mice (**Supplementary Figure 3**).

In order to examine whether the TCR signaling of *Cmas* KO T cells is changed, SP T cells of the thymus were stimulated with anti-TCR-β, followed by an anti-IgG crosslinking, and the Ca^2+^ mobilization was measured. In this experiment, we used SP cells of the thymus because the depletion of the *Cmas* allele is almost complete in this population (**Figure 2E**). The Ca^2+^ response in both CD4^+^ and CD8^+^ T cells was impaired in T cell-specific *Cmas* KO mice (**Supplementary Figure 4**). We concluded that the loss of sialoglycans on T cells affected the TCR signaling capacity.

### Complement C3 deficiency cannot rescue the T-cell defect of T cell-specific *Cmas* KO mice

**Figure 5:**
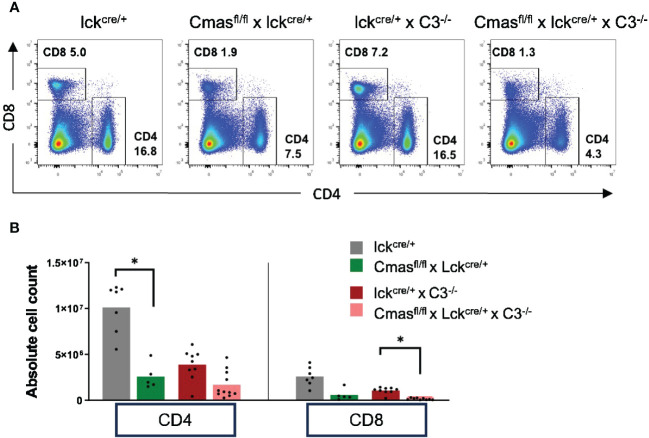
No rescue of the T-cell defect of T cell-specific *Cmas* KO mice by an additional complement C3 deficiency. *Cmas* ^fl/fl^ × lck-cre mice were crossed with complement C3-deficient mice. **(A)** Representative staining examples of splenic T cells of lck-cre controls, *Cmas* ^fl/fl^ × lck-cre mice, lck-cre × C3^−/−^ mice, and *Cmas* ^fl/fl^ × lck-cre × C3^−/−^ mice are shown. **(B)** CD4^+^ or CD8^+^ T cells of the spleen of the indicated mice were quantified, and cell numbers are given. n = 5–11 mice per group. Data are representative of nine individual experiments. *p ≤ 0.05 (Kruskal–Wallis test).

In order to determine the mechanism responsible for the reduced T-cell numbers in peripheral organs of T cell-specific *Cmas* KO mice, we first examined the role of complement in this pathway. α2,3-Linked Sia can bind to complement factor H to inhibit complement activation (4), and this inhibition could be missing in T cell-specific *Cmas* KO mice, explaining a T-cell loss by complement attack. In order to determine this, we crossed *Cmas* ^fl/fl^ × lck-cre mice with complement C3-deficient mice (C3^−/−^). When we compared the splenic CD4^+^ and CD8^+^ T-cell numbers of *Cmas* ^fl/fl^ × lck-cre × C3^−/−^ mice to the T-cell numbers of *Cmas* ^fl/fl^ × lck-cre mice, we did not find a rescue of the T-cell defect (**Figure 5**). We therefore conclude that complement attack is not the cause of the reduced T-cell numbers in the spleen of T cell-specific *Cmas* KO mice.

### T cells of T cell-specific *Cmas* KO mice show ongoing apoptosis

**Figure 6:**
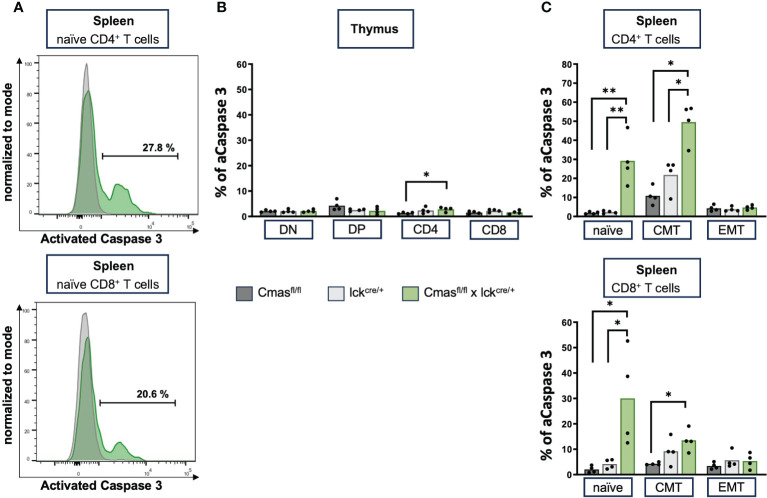
Splenic T cells of T cell-specific *Cmas* KO mice have high levels of activated caspase 3, indicating ongoing apoptosis. **(A)** Representative histogram overlays as examples of splenic T cells that are intracellularly stained with an antibody detecting activated **(A)** caspase 3. Shown are lck-cre mice (in gray) and *Cmas* ^fl/fl^ × lck-cre mice in green. **(B)** Bar diagram shows percentage of caspase 3 in double-negative (DN), double-positive (DP), CD4^+^, and CD8^+^ thymus T cells. **(C)** Bar diagram shows percentage of caspase 3 in splenic naïve, central memory (CMT), or effector memory (EMT) T cells (gated as in **Figure 3**). n = 4 mice per group. Data are representative of four individual experiments, each with one *Cmas* ^fl/fl^, lck^cre/+^, and *Cmas* ^fl/fl^ × lck^cre/+^ mouse. *p ≤ 0.05, **p ≤ 0.01 (Mann–Whitney *U*-test).

Next, we examined whether induction of apoptosis is responsible for the peripheral T-cell defect in T cell-specific *Cmas* KO mice. Spontaneous apoptosis was the main mechanism for the B-cell deficiency of B cell-specific *Cmas* KO mice (7). In order to analyze this, thymic T cells or splenic T cells were intracellularly stained with an antibody that recognizes activated caspase 3. Caspase 3 is an effector caspase that is activated by both intrinsic and extrinsic apoptosis pathways. In the thymus, no increased apoptosis levels were detected in DN, DP, or SP T cells of *Cmas* ^fl/fl^ × lck-cre mice, when compared to lck-cre controls (**Figure 6B**). However, in the spleen, both naïve CD4^+^ and naïve CD8^+^ T cells show a strongly increased proportion of cells with activated caspase 3. Central memory and effector memory T cells hardly showed this phenotype, but a significant increase of activated caspase 3 was also detected in CD4^+^ central memory T cells, when compared to the lck-cre controls (**Figures 6A, C**). This indicated an ongoing apoptosis in naïve peripheral T cells of T cell-specific *Cmas* KO mice, affecting mainly this peripheral T-cell population, which is strongly reduced in these mice. Supporting these data, naïve splenic T cells of *Cmas* ^fl/fl^ × lck-cre mice showed a higher exposition of the apoptosis marker phosphatidylserine on the outer layer of the cell membrane, indicating early apoptotic cells (**Supplementary Figure 5**). By this analysis, also a slightly higher number of apoptotic thymocytes of *Cmas* ^fl/fl^ × lck-cre mice were detected.

Peripheral T cells lacking sialoglycans may be deleted by cells bearing the asialoglycoprotein receptor (Ashwell–Morell receptor) that is involved in deleting desialylated platelets and clearing desialylated proteins. This receptor is expressed by hepatocytes in the liver (19, 20). We therefore examined whether T cells of *Cmas* ^fl/fl^ × lck-cre mice accumulate in the liver. However, we did not find higher T-cell numbers in the liver of *Cmas* ^fl/fl^ × lck-cre mice, when compared to lck-cre controls (**Supplementary Figure 6**).

### *Cmas* KO T cells have a strong competitive disadvantage compared to wild-type T cells

**Figure 7:**
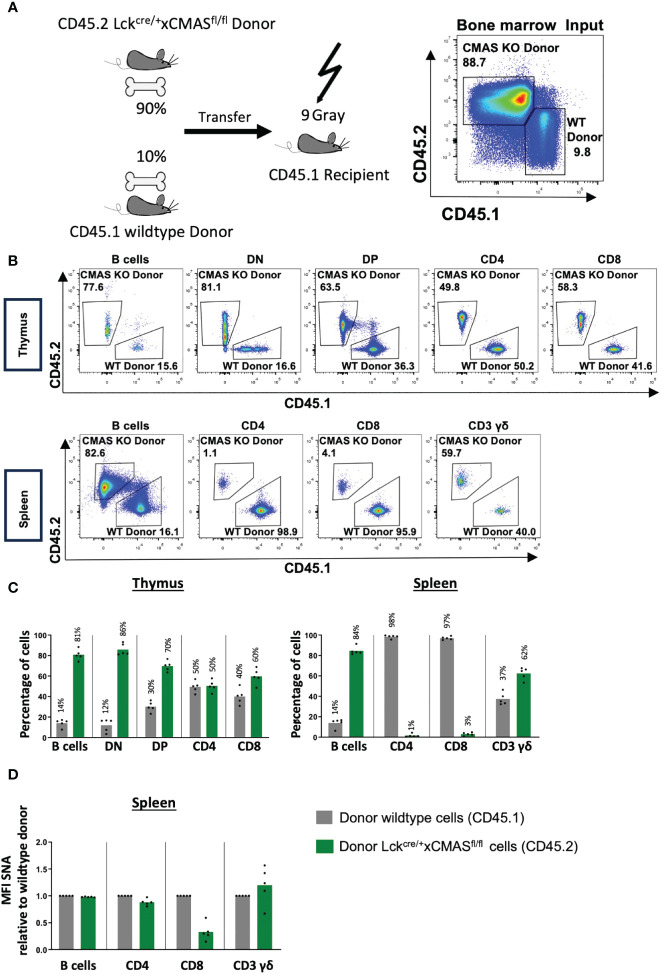
T cells of T cell-specific *Cmas* KO mice have a strong competitive disadvantage compared to wild-type T cells. **(A)** Bone marrow chimeras were generated as indicated in the scheme; 90% bone marrow of *Cmas* ^fl/fl^ × lck-cre (CD45.2) was mixed with 10% bone marrow of CD45.1 wild-type mice and i.v. injected into irradiated CD45.1 recipients. The bone marrow input is shown by CD45.1 vs. CD45.2 staining, shown here in representative dot plots. **(B)** Seven weeks after transfer thymic and splenic T-cell populations were analyzed by CD45.1 vs. CD45.2 staining. Representative staining is shown. **(C)** Thymic and splenic populations are quantified in relative numbers. Wild-type donor cells are identified as CD45.1, and *Cmas* ^fl/fl^ × lck-cre donor cells are identified as CD45.2. **(D)** α2,6-Linked sialic acid (Sia) on the cellular surface of splenic cells was detected by staining with the lectin *Sambucus nigra* agglutinin (SNA). The SNA mean fluorescence intensity (MFI) of the donor lck^cre/+^ × Cmas^fl/fl^ (CD45.2) cell populations was normalized to the mean of the MFI of the donor wild-type populations (CD45.1) (set as 1.0). Bars show means, and dots show individual mice. n = 5 mice per group. One representative out of two individual experiments is shown.

Since we observed a strong selection of wild-type T cells without deleted *Cmas* alleles in peripheral organs, we designed a mixed bone marrow (BM) transfer experiment with *Cmas* KO and wild-type BM cells to address their relative role in T-cell maturation. Since we expected a preference for wild-type precursors over *Cmas* KO precursor cells, we mixed the bone marrow of wild-type mice with the allelic marker CD45.1 and that of *Cmas* ^fl/fl^ × lck-cre mice with the marker CD45.2 in a ratio of 10% (WT) to 90% (KO). The mixed BM cells were transferred into sublethally irradiated recipient CD45.1 mice and analyzed 7 weeks after reconstitution (**Figure 7A**). The B-cell population served as a control population after the transfer. We observed a similar CD45.1/CD45.2 ratio of B cells in the thymus and spleen of the recipient mice 7 weeks after cellular transfer, as given as cellular input at day 0 (**Figures 7B, C**). Since we did not detect a much higher fraction of CD45.1 B cells than in the mixed BM donor cells, we conclude that the hematopoietic cells of the CD45.1 recipients were effectively deleted by irradiation; however, a small contribution of recipient cells cannot be excluded. For the thymocyte populations, we observed a similar ratio of 12% wild-type (CD45.1) cells in the DN population, as in the input. The percentage of wild-type cells in the thymus then constantly increased in further development in the DP to 30% and in the SP population to approximately 50% (**Figures 7B, C**). A strong competitive advantage of wild-type versus *Cmas* KO T cells was found in the spleen with a ratio of over 97% wild-type cells to less than 3% *Cmas* KO T cells. γδ T cells did not show this bias in cell numbers (**Figures 7B, C**). The remaining CD4^+^ T cells derived from the CD45.2 allele (*Cmas* KO) had an almost 100% expression level of sialic acid, as indicated by SNA staining, while the CD8^+^ T cells showed an expression level of 30% when compared to wild-type T cells (**Figure 7D**). This showed again the leakiness of the *Cmas* deletion in this mouse. The bone marrow chimeras demonstrated that in such a competitive situation, the *Cmas* KO αβ T cells are strongly out-competed by wild-type T cells. Thus, *Cmas* KO T cells are not selected or cannot survive in peripheral lymphatic organs.

## Discussion

The T cell-specific *Cmas* KO mice described here showed a normal T-cell development in the thymus, but a T-cell defect in the periphery. This is a similar finding as for B cells in B cell-specific *Cmas* KO mice but with a milder phenotype. The B-cell deficiency in the B cell-specific KO mouse was much more severe (7). We consider the milder T-cell defect to be a consequence of the incomplete deletion of the *Cmas* allele in the lck-cre-driven conditional KO mice. The incomplete deletion of the *Cmas* allele by lck-cre was detected by both lectin staining and genomic PCR analysis. Cre-mediated gene deletion is often not complete. Here, WT T cells with normal sialoglycan composition on the cellular surface were selected into the spleen and lymph nodes of T cell-specific *Cmas* KO mice. Splenic T cells that were found in T cell-specific *Cmas* KO mice had preferentially a memory phenotype. As this mouse is lymphopenic, this is not an uncommon finding. A homeostatic proliferation often occurs in lymphopenic mice. This may result in a peripheral T-cell population with memory-like T cells, as also found in other lymphopenic mouse lines (21). Peripheral naïve T cells need TCR-MHC-peptide contact for tonic signaling and survival. Memory T cells are much less dependent on this tonic signaling (22). TCR-MHC-peptide contacts may be affected by the loss of sialic acids, and therefore, this may contribute to a loss of naïve T cells of T cell-specific *Cmas* KO mice. Furthermore, it has recently been shown that sialic acids on the T-cell surface can inhibit CD28–ligand interactions (23). Therefore, naïve peripheral T cells of T cell-specific *Cmas* KO mice may also be strongly stimulated by this important co-stimulatory pathway, leading to a higher activation and a stronger effector memory phenotype. *Mgat1* ^fl/fl^ × Rag1-Cre mice with a deficiency in producing hybrid or complex sialylated N-glycans in T and B cells also showed a loss of naïve T cells and an activated phenotype in the spleen (8). Overall, as T cells in the thymus are found in normal numbers, our data point to a peripheral mechanism of the T-cell deficiency.

Lck-cre is known to be active in the DN stage in the thymus, and the proximal lck promoter present in these mice spares the γδ T cells from genetic deletion; therefore, no γδ T-cell phenotype was observed in the T cell-specific *Cmas* KO mice (18). We did not detect any signs of biased selection of T cells into the peripheral T-cell pool in T cell-specific *Cmas* KO mice. As the glycan composition on the T-cell surface changes during T-cell development, such changes in T-cell maturation or selection processes may have been expected (9). The staining of Vβ chains of the TCR with an antibody panel is not a very precise method to determine the TCR repertoire, but it served as a rough indicator of possible changes. Since no deviation from control mice was detected here, we did not analyze the TCR repertoire by other methods such as sequencing.

The bone marrow chimeric mice did show a clear competitive disadvantage of *Cmas*-deficient T cells when compared to wild-type T cells. There was a strong counterselection against *Cmas*-deficient T cells in the periphery. Thus, T cells without sialoglycan expression seem not to be able to be maintained in the periphery such as in lymph nodes or the spleen. Complement C3 deficiency was not able to rescue the T-cell defect of T cell-specific *Cmas* KO mice. In the B cell-specific *Cmas* KO, a small rescue effect by the C3 deficiency was observed. However, these mouse lines are difficult to compare, as in the *Cmas* ^fl/fl^ × mb1-cre mice a complete *Cmas* deletion occurred in peripheral B cells, which was not the case in the *Cmas* ^fl/fl^ × lck-cre mice. The T-cell conditional KO mice also had a high percentage of peripheral wild-type T cells, which, due to α2,3-linked Sia expression, should be protected against complement attack by the host.

The most likely mechanism for the T-cell defect of T cell-specific *Cmas* KO mice is the spontaneous apoptosis observed as high caspase 3 activation and Annexin-V exposure in naïve splenic T cells. Thus, we assume that peripheral T cells not expressing sialoglycans have a strongly reduced half-life, compared to wild-type T cells expressing sialoglycans on the surface. Strongly activated caspase 3 was also found in splenic B cells of B cell-specific *Cmas* KO mice (7). Spontaneous apoptosis was also described for CD8^+^ T cells in ST3Gal-I-deficient mice. In that mouse line, CD4^+^ T cells were not so much affected (10). This could be due to other sialyltransferases being involved in the sialoglycan formation of CD4^+^ T cells, either in α2,3-linkage or in other linkages. CD8^+^ T cells of ST3Gal-I-deficient mice were sensitive to PNA-induced apoptosis. PNA is a lectin that binds to β1,3-linked galactose lacking terminal Sia. As PNA also binds preferentially to *Cmas* KO T cells, an endogenous galactose-binding lectin could be involved in the mechanism observed here. A decrease of sialoglycans from the surface of lymphocytes has previously been detected as an “eat-me” signal for phagocytic cells (24). This process may be involved in the phenotype observed here, as well. As no accumulation of *Cmas* KO T cells in the liver was found, we conclude that the removal of desialylated T cells by the asialoglycoprotein receptor on hepatocytes (19, 25) is no major mechanism involved here. However, we cannot completely exclude the involvement of this pathway, as cellular elimination may happen quite fast.

As both B cells and T cells lacking sialoglycans are prone to apoptosis, this may indicate that the protection from apoptosis by sialoglycans is a general mechanism also for other cell types. Cancer cells have been described to upregulate sialoglycans on the surface (26). This may be a general strategy to provide protection from apoptosis. On this background, our findings underpin the potential of cancer treatment strategies focusing on interfering with sialoglycan expression. Such strategies are already the focus of current research (27–29).

## Data availability statement

The original contributions presented in the study are included in the article/**Supplementary Material**. Further inquiries can be directed to the corresponding author.

## Ethics statement

The animal study was approved by Regierung von Unterfranken, Würzburg, Germany. The study was conducted in accordance with the local legislation and institutional requirements.

## Author contributions

MS: Investigation, Validation, Writing – original draft. AL: Investigation, Writing – review & editing. MK: Investigation, Writing – review & editing. NS: Investigation, Writing – review & editing. SM: Writing – review & editing. FN: Writing – review & editing. AW: Writing – review & editing. MA: Writing – review & editing. RG-S: Writing – review & editing. AM-K: Funding acquisition, Writing – review & editing. LN: Conceptualization, Funding acquisition, Supervision, Writing – original draft.
