# Supplementary figures and images for: Sialic acids on T cells are crucial for their maintenance and survival
Source: Front Immunol. 2024 Jun 14;15:1359494. doi: 10.3389/fimmu.2024.1359494 (PMC11211268; doi:10.3389/fimmu.2024.1359494)

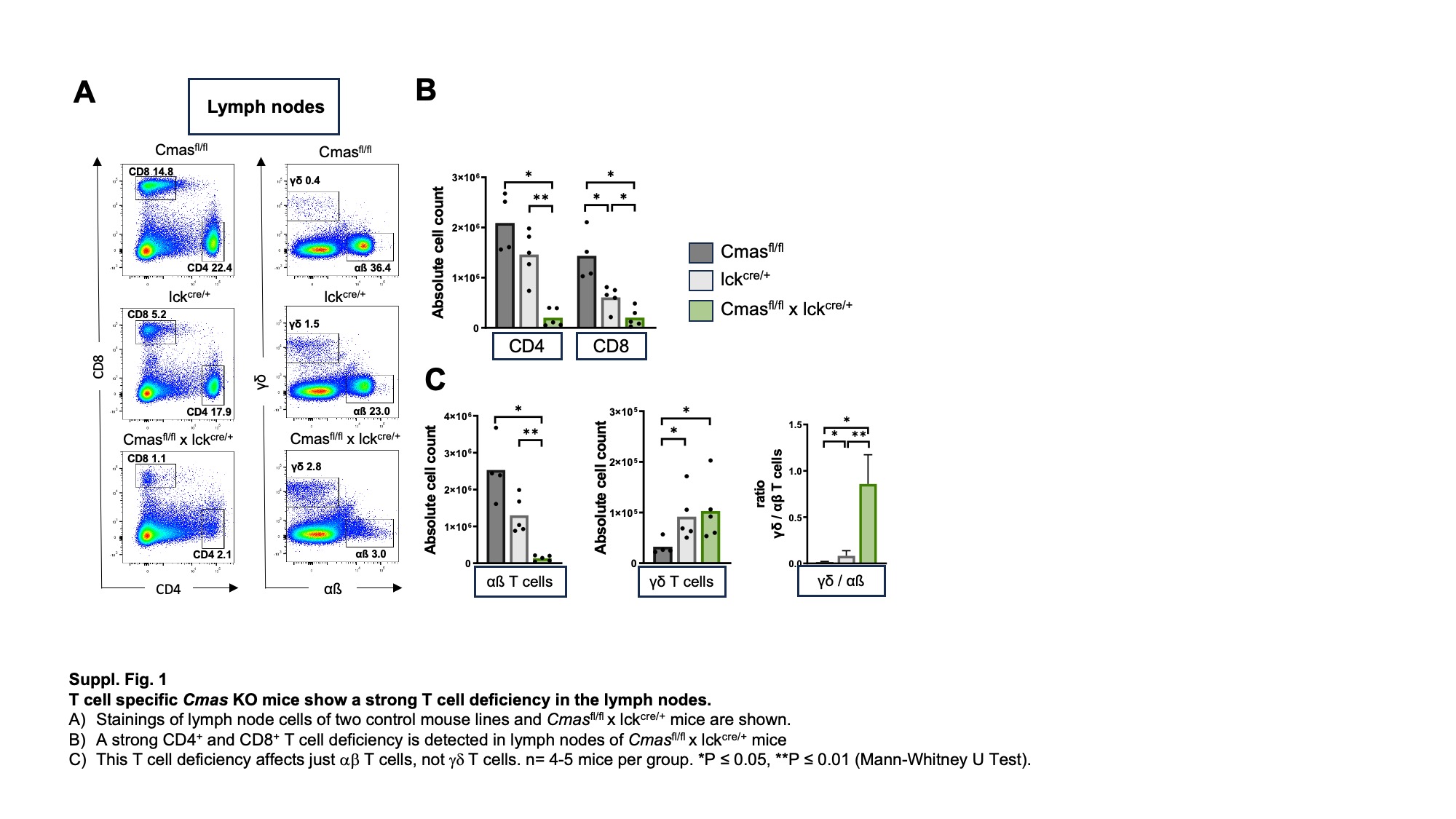

Supplement: Supplementary file 1 [file Image_1.jpg]

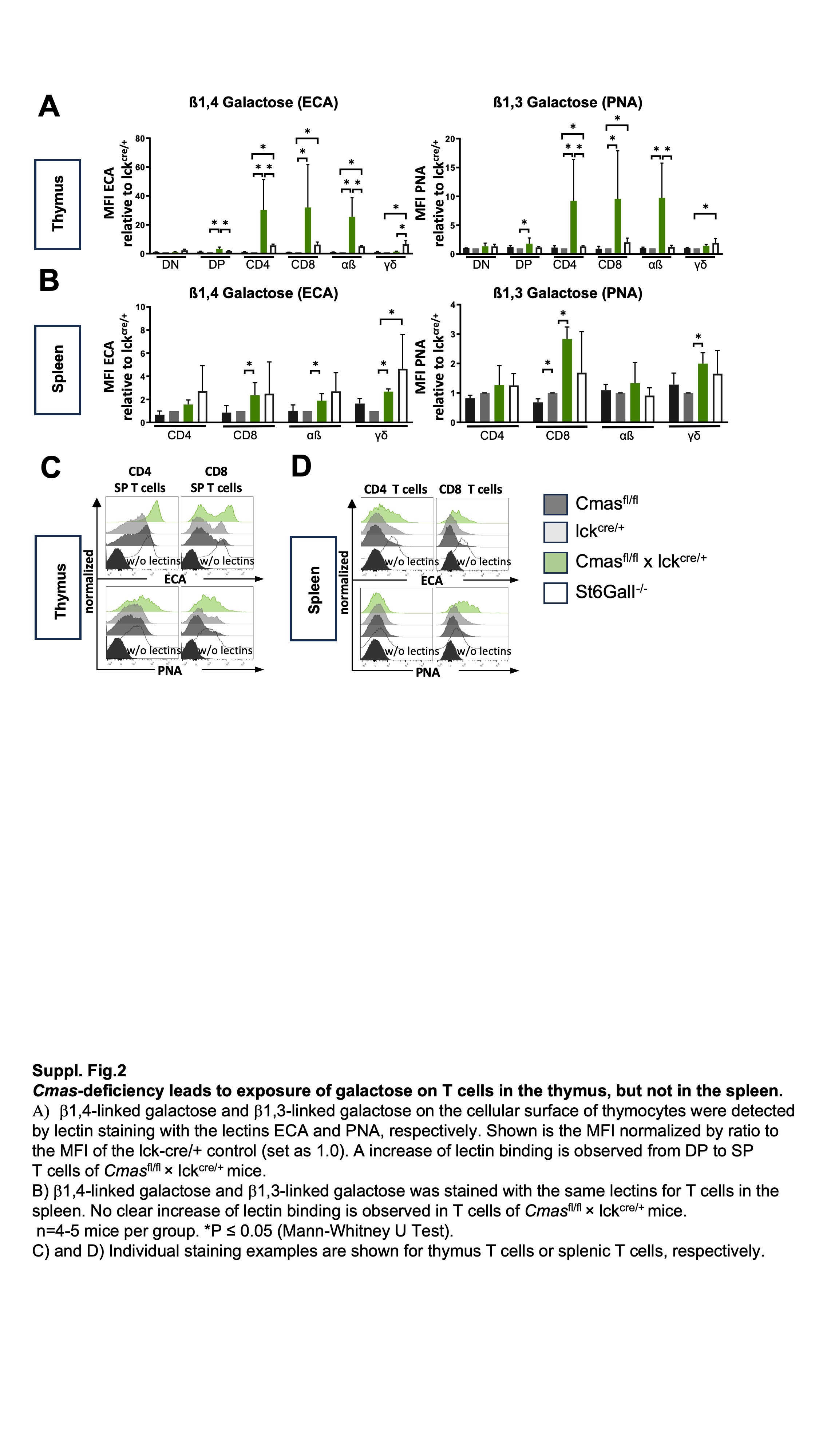

Supplement: Supplementary file 2 [file Image_2.jpeg]

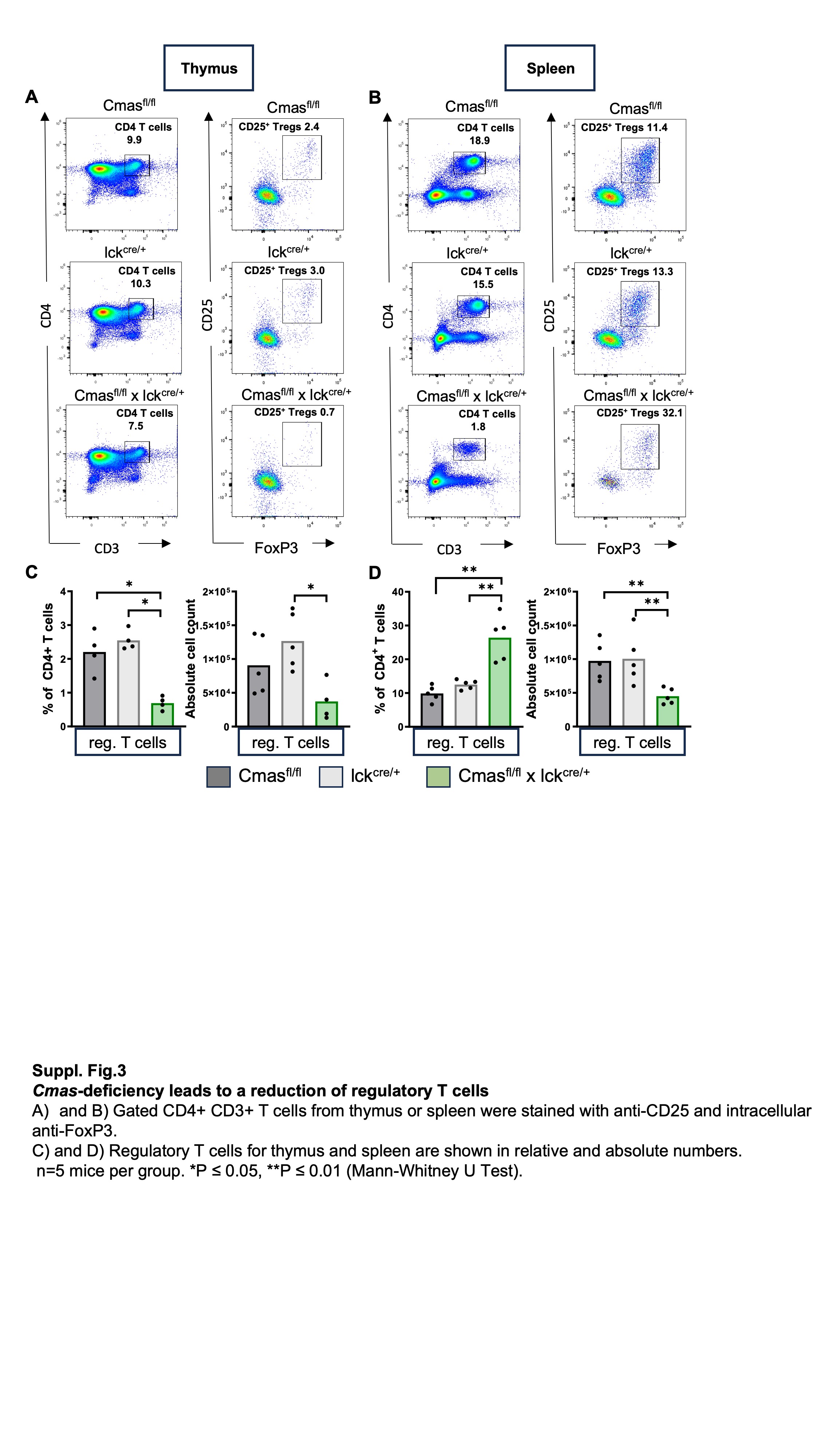

Supplement: Supplementary file 3 [file Image_3.jpeg]

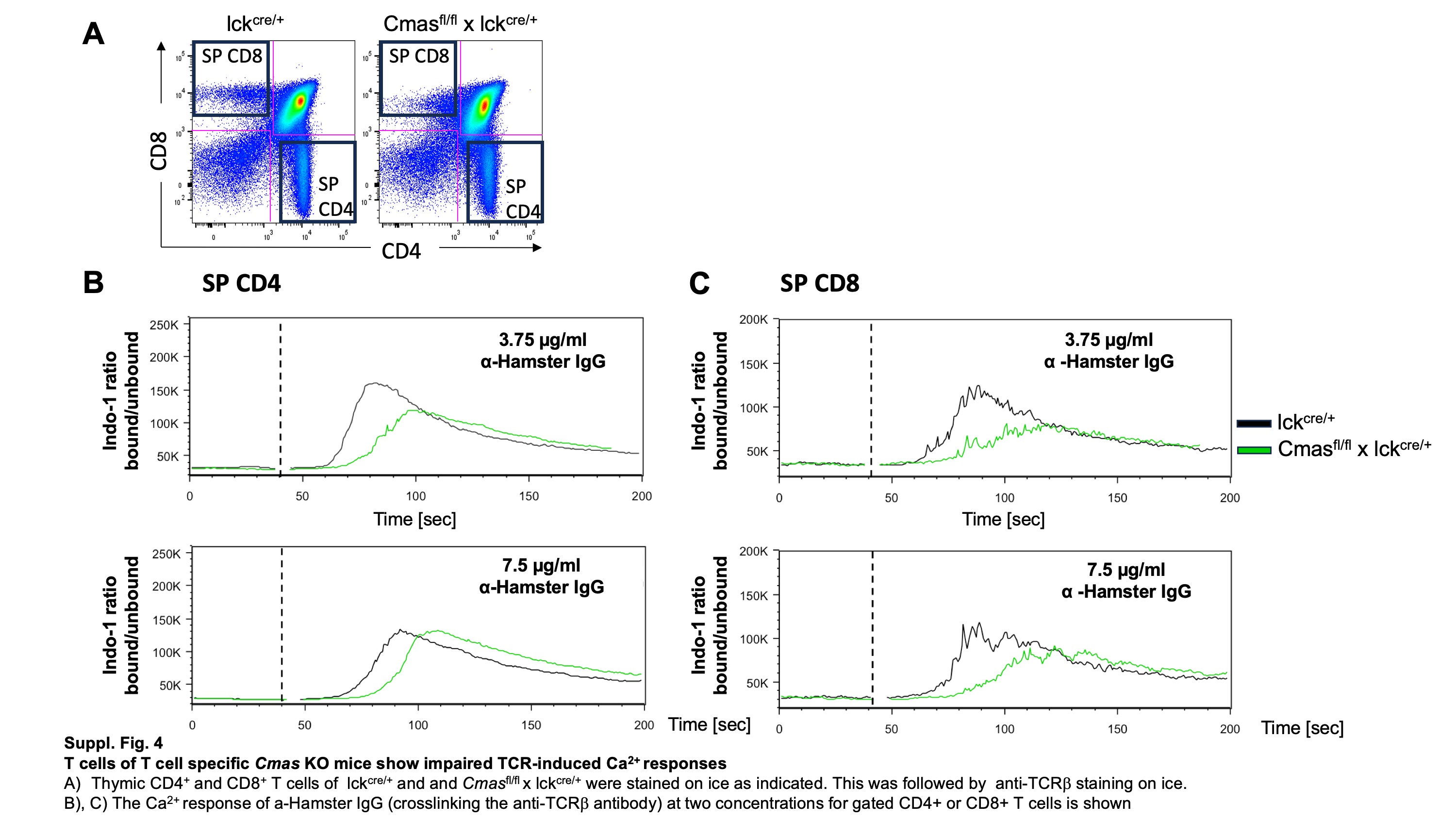

Supplement: Supplementary file 4 [file Image_4.jpeg]

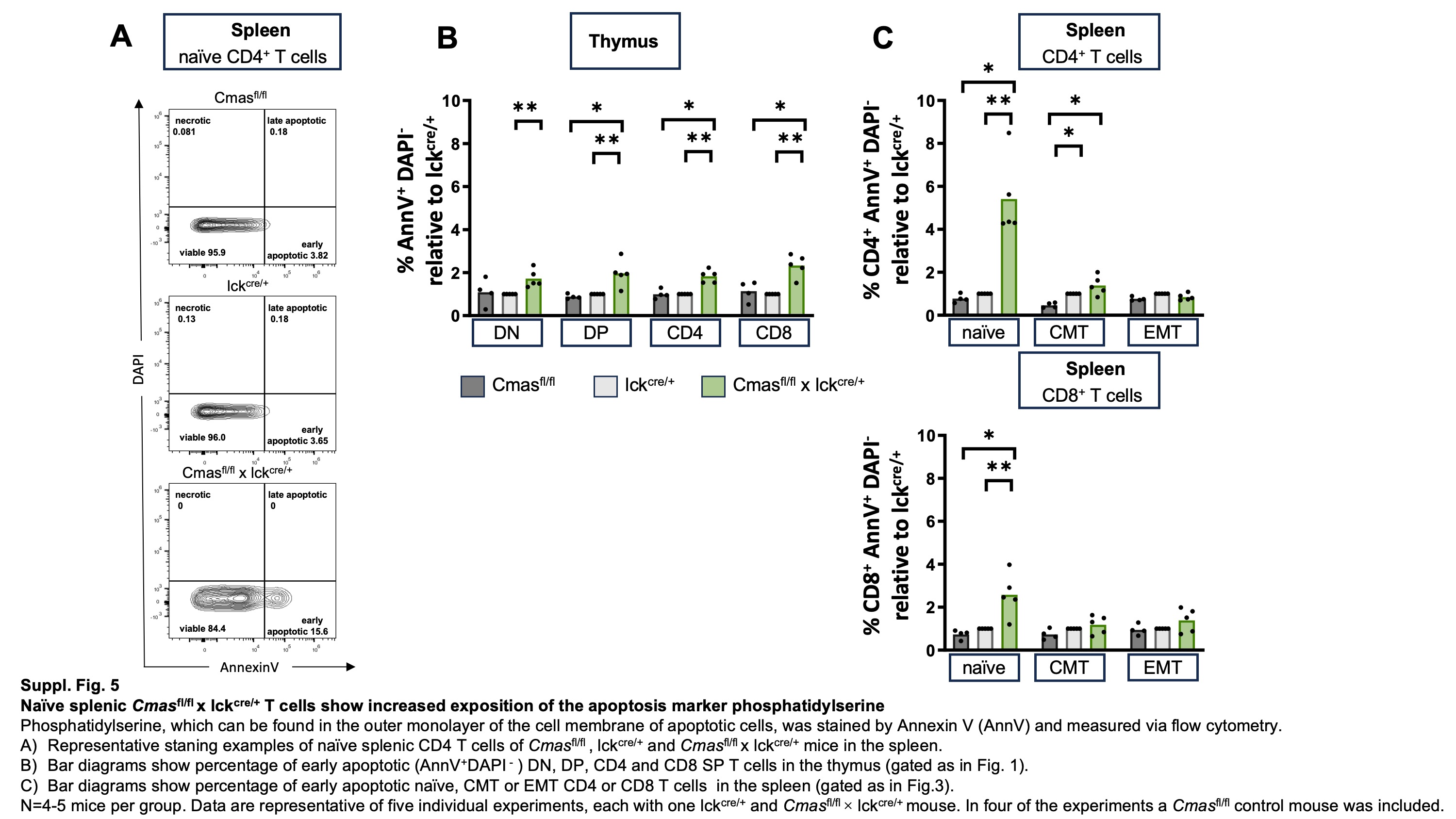

Supplement: Supplementary file 5 [file Image_5.jpeg]

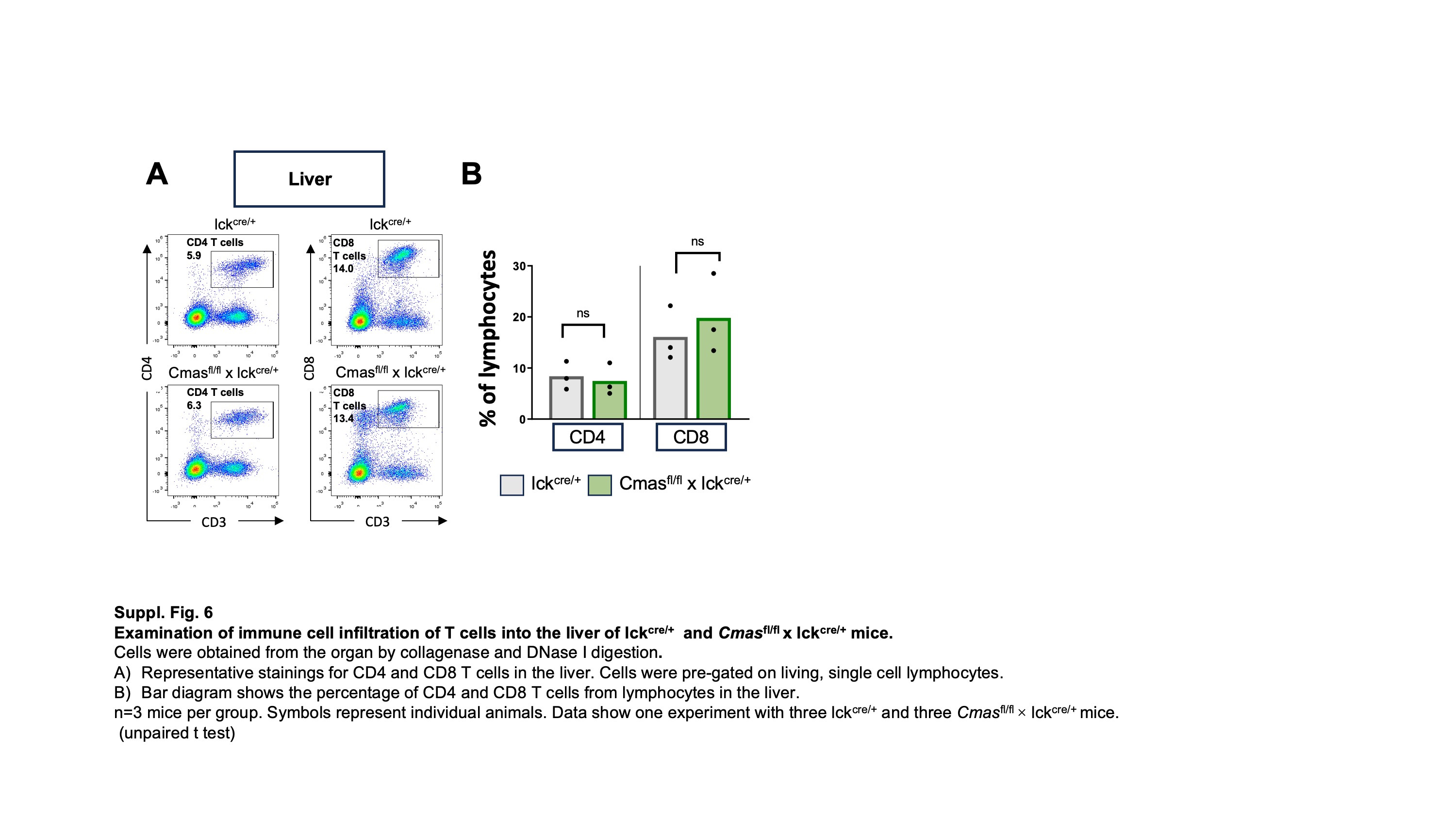

Supplement: Supplementary file 6 [file Image_6.jpeg]
